# Facial Expression Processing Across the Autism–Psychosis Spectra: A Review of Neural Findings and Associations With Adverse Childhood Events

**DOI:** 10.3389/fpsyt.2020.592937

**Published:** 2020-11-13

**Authors:** Celine Samaey, Stephanie Van der Donck, Ruud van Winkel, Bart Boets

**Affiliations:** ^1^Department of Neurosciences, Center for Clinical Psychiatry, KU Leuven, Leuven, Belgium; ^2^Department of Neurosciences, Center for Developmental Psychiatry, KU Leuven, Leuven, Belgium; ^3^Leuven Autism Research (LAuRes), KU Leuven, Leuven, Belgium; ^4^University Psychiatric Center (UPC), KU Leuven, Leuven, Belgium

**Keywords:** ASD, psychosis, facial expression processing, neuroimaging, childhood adverse events

## Abstract

Autism spectrum disorder (ASD) and primary psychosis are classified as distinct neurodevelopmental disorders, yet they display overlapping epidemiological, environmental, and genetic components as well as endophenotypic similarities. For instance, both disorders are characterized by impairments in facial expression processing, a crucial skill for effective social communication, and both disorders display an increased prevalence of adverse childhood events (ACE). This narrative review provides a brief summary of findings from neuroimaging studies investigating facial expression processing in ASD and primary psychosis with a focus on the commonalities and differences between these disorders. Individuals with ASD and primary psychosis activate the same brain regions as healthy controls during facial expression processing, albeit to a different extent. Overall, both groups display altered activation in the fusiform gyrus and amygdala as well as altered connectivity among the broader face processing network, probably indicating reduced facial expression processing abilities. Furthermore, delayed or reduced N170 responses have been reported in ASD and primary psychosis, but the significance of these findings is questioned, and alternative frequency-tagging electroencephalography (EEG) measures are currently explored to capture facial expression processing impairments more selectively. Face perception is an innate process, but it is also guided by visual learning and social experiences. Extreme environmental factors, such as adverse childhood events, can disrupt normative development and alter facial expression processing. ACE are hypothesized to induce altered neural facial expression processing, in particular a hyperactive amygdala response toward negative expressions. Future studies should account for the comorbidity among ASD, primary psychosis, and ACE when assessing facial expression processing in these clinical groups, as it may explain some of the inconsistencies and confound reported in the field.

## Introduction: Autism and Primary Psychosis as Distinct Yet Related Disorders

Autism spectrum disorder (ASD) and psychosis spectrum disorders are neurodevelopmental disorders characterized by impairments in social cognition. According to DSM-5, ASD is an early-onset disorder characterized by (1) difficulties in social interaction and communication, including deficits in socioemotional reciprocity and deficient nonverbal communicative behavior, and (2) the presence of restrictive and repetitive behaviors and interests and/or atypical sensory processing (1). Psychosis spectrum disorders are marked by positive symptoms, such as hallucinations and delusions, and negative symptoms, such as blunted affect and anhedonia. In addition, cognitive, affective, and social impairments may also be present (1). Psychosis spectrum disorders, which typically arise in young adulthood, include schizotypal personality disorder, delusional disorder, brief psychotic disorder, schizophreniform disorder, schizophrenia, schizoaffective disorder, and substance/medication-induced psychotic disorder. As these disorders are very heterogeneous in terms of symptoms, severity, and duration, we will adopt the term primary psychosis to represent all disorders included in psychosis spectrum disorders throughout this paper, except when the evidence is specific to individuals with a particular diagnosis.

Despite clear differences between ASD and primary psychosis, in particular regarding age of onset, historically, both disorders have been considered as closely related pathologies (2, 3). In fact, DSM-II (4) did not differentiate between the two disorders and listed autistic behavior as one of the criteria for childhood schizophrenia (5). Research from the 1970s onwards showed that a reliable distinction could be made between ASD and primary psychosis (6); yet, studies continuously demonstrated a considerable overlap between these two conditions (7). More recently, research has again been focusing on evidence for the connection between ASD and primary psychosis. Recent meta-analyses revealed a significant epidemiological association between both disorders [(8, 9); for an umbrella review, see (10)]. More specifically, the prevalence of primary psychosis in individuals with a childhood ASD diagnosis is significantly higher than in controls (odds ratio = 3.55) (9), with an estimated weighted pooled prevalence of up to 9.5% of primary psychosis in individuals with ASD (8). Vice versa, the prevalence of comorbid ASD in primary psychosis (ranging from 3.4 to 52%) is also higher than in the general population (9). A recent study revealed significantly increased rates of autistic traits in patients with psychosis, with 6.5% scoring above clinical cutoff; yet, there was no significant difference between people with familial risk and healthy controls (11).

Notably, both ASD and primary psychosis are also associated with an increased prevalence of adverse childhood events (ACE) [(12, 13); for reviews, see (14–16)]. On the one hand, ACE, such as physical, sexual, or emotional abuse and physical or emotional neglect, have consistently been shown to be related to the onset of psychotic symptoms and the development of primary psychosis (15, 16). On the other hand, children with ASD may be more prone to experience ACE due to their social and communicative difficulties and their struggles with daily life skills (17, 18). Furthermore, heritability rates for ASD and primary psychosis are high and both disorders share overlapping genetic mechanisms (7, 19, 20).

In addition to overlapping epidemiological, environmental, and genetic components, both disorders display multiple endophenotypic similarities. At a behavioral and cognitive level, individuals with ASD and primary psychosis show overlapping symptomatology, impeding the differentiation between psychosis and ASD symptoms and hampering differential diagnosis [for a review and meta-analysis, see (21)]. In both disorders, social interaction, and communication deficits may be present, as well as theory of mind, mentalizing, and general social–cognitive functioning impairments, such as the lack of socioemotional reciprocity (7, 21, 22). In addition, individuals with ASD often display positive psychotic experiences, while patients with primary psychosis may also show restricted interest, mental inflexibility, and reduced social attunement (7).

At a neural level, similar structural and functional atypicalities have been reported in both disorders, in particular pertaining to social brain areas. For example, both disorders show lower gray matter volumes in the right parahippocampal gyrus, posterior cingulate, putamen, insula, and left thalamus (23). This finding is supported by reduced cortical thickness and surface area in children and adolescents with ASD and early-onset first-episode primary psychosis, which may serve as a potential early neurodevelopmental mechanism in the pathogenesis of both disorders (24). Furthermore, volume loss in prefrontal areas and the temporal–parietal junction, volume gains in the caudate, and reduced fractional anisotropy values (indexing reduced structural connectivity) have been reported in both ASD and childhood-onset primary psychosis (7, 9). Besides these structural similarities, functional similarities have also been reported in individuals with ASD and primary psychosis (25). During social cognition tasks, both groups display reduced activation in medial prefrontal areas, yet this deficit is larger in individuals with ASD. Moreover, individuals with ASD and primary psychosis both exhibit amygdala hypoactivity and reduced activity in the superior temporal sulcus (STS) during social cognition tasks (25).

One of the key processes underlying impairments in social interaction and communication deficits common to ASD and primary psychosis may be facial expression processing. Indeed, accurate facial expression processing is crucial for social communication, as facial expressions convey important social cues and constitute a large portion of nonverbal communication. Consequently, impairments in facial expression processing very likely contribute to poor psychosocial functioning in psychiatric disorders including ASD (26) and primary psychosis (27). In a similar vein, impaired facial expression processing may impact psychosocial functioning in individuals exposed to adversity (28, 29). The development of adequate facial expression processing is largely driven by visual experience during childhood (28, 30). Faces provide important cues about the emotional state of the interacting partner, in particular the primary caregiver, and infants learn to read and interpret this emotional information. In the case of unsafe social environments, children may become hypersensitive to (facial) cues signaling threat, and this threat signaling may subsequently generalize to more ambiguous emotional cues (30). Therefore, children exposed to atypical emotional environments, such as ACE, are expected to show atypical facial expression processing.

Thus far, the face processing literature has typically been focusing rather exclusively on isolated syndromes, thereby ignoring possible commonalities and associations among various syndrome clusters. Accordingly, the present narrative review aims to adopt a broader more overarching perspective in order to provide a concise overview of commonalities and differences in the current neuroimaging literature on facial expression processing in ASD and primary psychosis, as well as to provide insight into the impact of ACE on facial expression processing.

The studies included in this narrative review were identified through a series of literature searches in online databases (*PubMed, ScienceDirect, Web of Science*) and *Google Scholar* for papers published within the last 20 years (2000–2020) using a combination of the following keywords: auti^*^, autism, ASD, psychosis, schizo^*^, trauma, abuse, maltreatment, advers^*^, *emotional face processing, emotion processing, facial expression processing, neuroimaging, EEG*, and *fMRI*. Additional studies were encountered in the reference lists of selected studies.

As evidenced by the different keywords used in our literature search to define ACE, definitions of ACE vary greatly, ranging from experiences of poverty and neglect to physical, emotional, and sexual abuse. Likewise, there exists a multitude of operationalizations to characterize ACE. For instance, ACE can be assessed prospectively or retrospectively, and these measures are often used interchangeably, even though a recent meta-analysis (31) confirmed that there is only very limited agreement between them. Furthermore, ACE are generally measured via child services reports or via questionnaires and interviews. The latter show a large variability in the type of adversity included and can either be self-report or parent-report [for reviews, see (32, 33)]. As a result, ACE are often recorded differently in different studies, thereby reducing comparability and generalizability of the reported findings. For the present qualitative review, we applied a rather broad definition of ACE, including experiences of neglect and physical, emotional, and sexual abuse. Similarly, facial expression processing serves as an umbrella term, covering various operationalizations to pinpoint individual differences in the sensitivity for processing particular facial emotional cues. This process can be assessed via explicit behavioral tasks (i.e., with explicit attention to the emotional features) or via implicit measures (e.g., via eye-tracking, neuroimaging, autonomic nervous system reactivity, etc.). Furthermore, a large variety of behavioral facial expression processing tasks can be administered, such as labeling facial expressions, matching emotional faces, discriminating or differentiating between different emotions, judging the intensity of emotional expressions, detecting a specific facial expression in a series of faces displaying different emotions, etc. In addition, low-level stimulus features (e.g., spatial frequency) and emotion-specific stimulus dimensions (e.g., intensity, valence) also impact on the emotion processing and can possibly determine whether group differences are observed (34). For the present report, we focused upon behavioral face processing indices and neural face processing mechanisms as measured via electroencephalography (EEG) and functional magnetic resonance imaging (fMRI).

## Neural Mechanisms of Facial Expression Processing

Typically, facial expression recognition develops and improves with age (35, 36), but the developmental trajectories are emotion-specific (35, 37). Happiness, for example, is recognized the earliest, fastest, and most accurate (38), reaching adult levels at 5 to 6 years of age (36), whereas the recognition of anger, for instance, steeply improves (39, 40), with a clear increase in sensitivity from adolescence into adulthood (41).

In addition to behavioral improvement, facial expression processing also matures from early infancy to adolescence at the neural level (42–44). Extensive fMRI research has delineated a core face processing network consisting of the occipital and fusiform gyri [also known as the occipital and fusiform face area (OFA and FFA), respectively] (45) and the posterior STS (46). More specifically, invariant aspects of faces, such as facial identity and gender, are mainly processed by the ventral occipito-temporal cortex (OFA and FFA), whereas dynamic and variant aspects of faces, such as eye gaze and expression, are mainly processed by the posterior STS (47). This core face processing system is activated when processing expressive faces (48–50), along with areas of an extended face processing network involving visual, temporo-parietal, prefrontal, and subcortical areas (51–54) to extract meaning from these faces (55, 56). The amygdala, for instance, plays a crucial role in processing expressive faces and allows prioritizing of processing emotionally salient stimuli (49, 53, 57). In addition to the generally increased neural activation during emotion processing, there also seems to be a differential response pattern for specific emotions. For instance, the limbic system is especially sensitive to fearful and, to a lesser extent, happy expressions, whereas the insula shows increased activation when processing disgusted faces and, to a lesser extent, angry faces (52).

Unlike fMRI, EEG has a very high temporal resolution and is therefore optimal for studying the temporal course of facial expression processing (58). Event-related potentials (ERPs) have been widely used to investigate the neural mechanisms supporting facial expression processing in different populations, including individuals with ASD [for reviews, see (59–62)] and individuals with primary psychosis (63, 64). Research has pinpointed the N170, an ERP component with a negative peak occurring ~170 ms after stimulus onset, as a consistent marker of face processing as it is more responsive to faces than objects (58, 65, 66). Despite conflicting reports in the literature, a meta-analysis by Hinojosa et al. (65) found that the N170 is especially sensitive to expressive faces, as its amplitude is larger in response to expressive faces compared with neutral faces. Yet, evidence from a recent integrative review did not support the presence of a distinctive discrimination pattern between the different facial emotions, apart from the differential encoding of emotional expressions from a neutral expression (67). In addition to the N170, a broader range of ERP components—which reflect different stages of neural processing—has been found to be modulated by facial expressions. Other authors have shown that both the latency (68) and amplitude (69, 70) of the P100 are influenced by facial expressions, especially by fear. Likewise, sadness (71), fear, and anger (70) have been found to elicit larger late positive potential responses as compared with neutral faces [for a review, see (72)]. Furthermore, anger (73) and other basic expressions (74) have been shown to evoke larger early posterior negativity.

Facial expression processing is thus guided by a complex, interconnected network of neural structures (49, 52). In the following sections, we will provide a brief summary of findings from neuroimaging studies investigating facial expression processing in ASD and primary psychosis (see Table 1 for an overview). Considering the extensiveness of the neuroimaging literature and the scope of this paper, for the fMRI data, we will mainly focus on the most commonly investigated brain areas during facial expression processing, more in particular the core and extended face processing network, as well as the social brain areas.

**Table 1:** Overview of the reported neural alterations in individuals with ASD and primary psychosis when processing facial expressions, in comparison with typically developing individuals.

	**ASD**	**Primary psychosis**
**fMRI STUDIES**		
Amygdala	**Activation** Hypoactivation **Functional connectivity** Altered connectivity between amygdala and -Medial prefrontal cortex -Fusiform gyrus -STS	**Activation** Hypoactivation Hyperactivation **Functional connectivity** Altered connectivity between amygdala and -Precuneus -Temporo-parietal junction
Fusiform gyrus	**Activation** Hypoactivation	**Activation** Hypoactivation
STS	**Activation** Hyperactivation	
Other brain regions	**Activation** Hypoactivation in -Temporo-parietal junction -Medial prefrontal cortex	**Activation** Hypoactivation in -Hippocampal region -Anterior and middle cingulate cortex -Dorsolateral and medial frontal cortex -Insula -Thalamus -Caudate -Lentiform nucleus -Putamen -Basal ganglia Hyperactivation in -Left middle occipital gyrus -Cuneus -Left precuneus -Inferior parietal lobule -Precentral gyrus -Right middle frontal gyrus -Dorsolateral prefrontal cortex
**ERP STUDIES**		
P100	**Latency** Delayed response **Amplitude** Reduced amplitude	**Amplitude** Reduced amplitude
N170	**Latency** Delayed response **Amplitude** Reduced amplitude	**Amplitude** Reduced amplitude
Other ERP components	**N300 latency** Delayed response **N400 latency** Faster response **N400 amplitude** Reduced amplitude **NSW amplitude** Less differentiated amplitude in function of facial expression	**N250 amplitude** Reduced amplitude **P300 amplitude** Reduced amplitude
**FREQUENCY-TAGGING EEG STUDIES**		
	**Oddball response** Reduced neural sensitivity for angry and fearful faces	

*STS, superior temporal sulcus; NSW, negative slow wave*.

### Facial Expression Processing in ASD

#### Behavioral Findings

An abundance of behavioral studies has investigated the facial expression processing abilities of individuals with and without ASD, yielding, however, mixed and inconsistent results in terms of group differences (34, 75–78). Most studies suggest a general emotion processing deficit in ASD as compared with healthy controls (79–82), yet some researchers only find difficulties with specific—mostly negative—emotions (83–86). Fear has been shown a difficult to recognize expression for individuals with ASD, especially for adults (77, 87). In addition, some studies also reported reduced recognition abilities for positive emotions, such as surprise (84) and happiness (83). Generally, though, individuals with and without ASD perform equally well for happy facial expressions (84–86). In contrast to the findings described before, other studies have reported intact facial expression recognition in ASD (88–90). Intact recognition abilities may indicate the use of verbally mediated or cognitive compensatory mechanisms in ASD to recognize facial expressions, whereas this process is more automatic in typically developing individuals (75). Hence, the interpretation of explicit emotion processing results can be impeded due to mechanisms beyond facial expression processing *per se*. In addition, given that a considerable degree of the conflicting findings on facial expression recognition can be attributed to task demands, the highly variable behavioral results may reflect the variability and limited sensitivity of certain behavioral measures (75).

#### fMRI Research

To overcome these impediments of behavioral measures and to understand the neural basis of facial expression processing in ASD, many researchers have turned to neuroimaging measures, such as fMRI. However, also fMRI studies generally fail to draw consistent conclusions on the brain anomalies of individuals with ASD. For example, although a previous review (91) and a meta-analysis (92) reported a generally hypoactivated fusiform gyrus in individuals with ASD as compared with healthy controls during facial expression processing, a more recent meta-analysis (93) showed similar activation patterns in both groups. In a similar vein, in contrast to the previously reported hyperactivation of the STS in individuals with ASD as opposed to healthy controls (92, 94), the meta-analysis of Aoki et al. (93) indicated no group differences in STS activation. However, differences in the applied analysis method might (partially) account for the differences in results: the more recent meta-analysis was conducted using seed-based *d* mapping, whereas Di Martino et al. (94) and Philip et al. (92) applied the activation likelihood estimation approach. Moreover, the accumulated number of included empirical studies may also have contributed to the different results.

Pertaining to the amygdala, the amygdala theory of autism postulates that atypicalities in the amygdala are at the root of the characteristic social deficits of individuals with ASD (95). Hypoactivation of this region has, indeed, frequently been found (93, 94), especially when processing fearful faces (96, 97). In addition, substantial evidence points toward a dysfunctional connectivity between the amygdala and the medial prefrontal cortex [(98); for a review, see (99)], possibly resulting in the socioemotional difficulties in ASD. Indeed, as these brain regions are employed when perceiving and assessing socioemotional information, the atypical connectivity might be associated with more severe social difficulties and less social orienting (100). Furthermore, individuals with ASD also display altered functional connectivity between the amygdala and other areas of the social brain, such as the fusiform gyrus and STS, when implicitly processing fearful faces (96) or explicitly processing angry and happy faces (101).

While Ciaramidaro et al. (102) reported differential activation in the temporo-parietal junction and the medial prefrontal cortex—two regions crucially involved in theory of mind processing (103)—in individuals with ASD when processing expressive faces, no general group difference in brain activity has been found in these regions on the basis of a formal meta-analysis (93).

Given that neural activity in social brain regions can be modulated by experimental parameters (91, 92, 94), the methodological variability across studies might contribute to the inconsistent findings (104). For instance, activation in the fusiform gyrus can be influenced by the degree of visual attention to the expressive faces and to the eyes, as evidenced by similar activation patterns in individuals with and without ASD when cues explicitly guided the visual attention of participants with ASD toward the faces (105, 106). These results have been interpreted in light of the social motivation theory of ASD (100), suggesting that hypoactivity in the fusiform gyrus in the absence of guiding cues might reflect the lack of motivation to attend to salient expressive facial features (92). Similar effects have been reported for the amygdala, with enhanced amygdala activity when attention is oriented toward the eyes of the faces (92, 107). In addition, modulatory effects of task demands on neural activation during facial expression perception have also been reported (91, 92, 94). For example, explicit vs. implicit facial expression processing elicit differential responses in the amygdala, fusiform gyrus, or STS, both in individuals with (102, 108) and without ASD (48). Furthermore, also stimulus characteristics, such as intensity of the expressions (83, 96), familiarity of the faces (109), or static vs. dynamic expressions (54), have been found to influence the neural responses.

#### EEG Research

In addition to fMRI, EEG is a suitable method for ASD research, given its noninvasive nature and the nonrequirement of verbal or motor responses (110). ERPs have been widely used to investigate perceptual mechanisms supporting face and emotion processing abilities in individuals with and without ASD (60, 62). However, up until now, ERP studies have also generally failed to draw consistent conclusions on facial expression processing in ASD (59, 75). Similar ERP patterns in children, adolescents, and adults with and without ASD have been found (111–114), yet others have reported differences. Differences in the latency and/or amplitude of early ERP components, such as P100 (81, 115), N170 (113, 116–118), and N300 (119), for different facial expressions have frequently been found in individuals with ASD, suggesting reduced or delayed facial expression processing. However, anomalies have also been reported in later ERP components [e.g., N400 (117, 120) or negative slow wave (119)], which are believed to be more related to emotion categorization than to affective processing (62, 67). Differences in ERP components, particularly in the N170, between both groups have often been reported for fearful faces (117–119).

Recently, the N170 has been put forward as a possible biomarker of the underlying neural face processing deficits in individuals with ASD (61). However, standard ERP techniques are seriously limited in objectively defining components in the time domain and quantifying responses of interest at the individual subject level (121). Moreover, observed group differences in N170 latency and/or amplitude could merely reflect a slower general processing of social stimuli in individuals with ASD (44, 116, 121), or they could be caused by carryover effects from changes in the amplitude and/or latency of the immediately preceding P100 component (122). In addition, atypicalities in this ERP component (i.e., delayed and/or reduced response) may not be autism-specific: similar atypical N170 responses have been observed in other neurological and psychiatric disorders, in particular in primary psychosis, hence, possibly rather indicating facial expression processing dysfunction as a symptom of these diagnoses, than a disorder-specific deficit (58).

To meet some of the methodological limitations of the standard ERP approach, a novel fast periodic visual stimulation frequency-tagging EEG approach was recently introduced in the autism field [see (123) for a review]. This novel tool offers great advantages in terms of objectivity in the identification and quantification of selective responses of interest in the frequency domain of the EEG spectrum, as well as high sensitivity (i.e., high signal-to-noise ratio). A pioneering study by Van der Donck et al. (124, 125) applied a frequency-tagging EEG oddball paradigm to assess the neural sensitivity for rapid changes in emotional expressions, revealing reduced neural discrimination responses for fearful and angry faces in children with ASD and predicting clinical status with an 87% accuracy at the individual level.

Although the previously described findings provide insights in the underlying neural nature of facial expression processing difficulties in ASD, multimodal imaging (i.e., integrating imaging methodologies) might advance our understanding of these mechanisms even further. In recent years, this powerful approach has increasingly been applied to study the main characteristics of ASD (126, 127). However, to date, the number of studies investigating facial expression processing in ASD using multimodal imaging is limited [for example, see Corbett et al. (128)]. In particular, complementing EEG and fMRI face processing studies with white matter structural connectivity diffusion-weighted MRI tractography might be a promising avenue for ASD research, as reduced white matter integrity has been observed in the occipito-temporal cortex (129) and specifically along the inferior longitudinal fasciculus (130, 131), a tract which is crucially involved in neural face processing. Altogether, despite the inconsistencies in the neuroimaging literature regarding facial expression processing in individuals with and without ASD, overall, both fMRI and EEG findings seem to indicate a differential, possibly reduced, facial expression processing ability in individuals with ASD.

### Facial Expression Processing in Primary Psychosis

#### Behavioral Findings

In contrast to the mixed and inconsistent findings of behavioral facial expression processing in ASD, behavioral studies have consistently reported social cognition deficits in individuals with primary psychosis compared with healthy controls, including deficits in facial expression perception (27). Impaired facial expression perception in primary psychosis has been demonstrated across a variety of tasks, including emotion identification and higher-level social judgments, and is especially apparent for negative emotions. Moreover, these deficits are present early in the course of psychosis, as they have been reported in first-episode psychosis, and have been related to functional outcomes (27, 132).

#### fMRI Research

Overall, individuals with primary psychosis have been shown to display hypoactivity along the core face processing network, more specifically the fusiform gyrus, as well as structural abnormalities and hypoactivity along the extended face processing network. Furthermore, there is evidence of some compensatory hyperactivity along areas that do not typically belong to the social brain network.

During facial expression perception, individuals with primary psychosis display reduced activity in early visual processing regions, the amygdala/hippocampal region, anterior cingulate cortex, dorsolateral frontal cortex, and medial frontal cortex. A recent meta-analysis revealed decreased activity in two clusters during facial expression processing: one extensive cluster including the right ventrolateral prefrontal cortex, cingulate, insula, amygdala, thalamus, caudate, lentiform nucleus, and putamen and a cluster with the anterior and middle cingulate cortex (133). Similarly, Kret and Ploeger (134) described reduced activity in the fusiform gyrus, amygdala, and basal ganglia, as well as a reduced functional connectivity between the amygdala and precuneus and temporo-parietal lobe. Activity in the bilateral fusiform gyrus and right superior frontal gyrus is also significantly impaired in individuals with primary psychosis (135). These findings seem to depend on task demands however, as, for instance, hypoactivation in the fusiform gyrus is mainly found during implicit tasks (136). Yet, atypical amygdala activity is reported both in implicit and explicit tasks, indicating a robust impairment in individuals with primary psychosis (135).

Pertaining to atypicalities in amygdala functioning, functional differences during facial expression processing have consistently been reported, especially in response to negative emotions, yet the reported direction of these abnormalities is highly inconsistent. Whereas meta-analyses have mainly demonstrated reduced amygdala activity (133–135), some studies also revealed increased amygdala activity in individuals with primary psychosis (137).

These inconsistent results in terms of hypo- vs. hyperamygdala activity can be explained by differences in the applied baseline contrast used to calculate responses to expressive faces, i.e., whether expressive faces are contrasted vs. a neutral face or whether they are contrasted vs. a nonface baseline. In line with the aberrant salience hypothesis (138), it is hypothesized that individuals with primary psychosis assign too much emotional salience to neutral stimuli, thus also to neutral faces (136). Studies assessing neural activity in response to neutral faces have indeed found increased activity in amygdala, as well as in prefrontal, cingulate, and parahippocampal regions, in individuals with primary psychosis as compared with healthy controls (137, 139). Consequently, the relative hypoactivity in amygdala found in many studies assessing primary psychosis may well be a methodological artifact induced by contrasting expressive faces with faces with a neutral expression. This particular interpretation was corroborated by a formal meta-analysis of amygdala activation in individuals with primary psychosis, identifying methodological heterogeneity as an explanatory factor of the inconsistent findings (140). In general, bilateral amygdala activity in response to expressive faces was significantly reduced, but this hypoactivity was only apparent in studies using the expressive minus neutral face contrast. This finding suggests that the true difference in amygdala activity between individuals with primary psychosis and healthy controls might be an elevated amygdala response toward emotionally neutral stimuli rather than decreased activity to emotional faces (140).

In contrast to the underactivity in the emotion processing areas described above, individuals with primary psychosis often demonstrate increased activation in other areas not typically associated with facial expression processing. Recent reviews revealed increased activity in the left middle occipital gyrus, cuneus, left precuneus, inferior parietal lobule, precentral gyrus, right middle frontal gyrus, and dorsolateral prefrontal cortex (133, 134, 136). Hyperactivation in these areas could be interpreted as a compensatory mechanism for the disrupted neural activity during behavioral performance on explicit facial expression processing tasks in primary psychosis.

Generally, neural atypicalities during facial expression processing in individuals with primary psychosis are robust and have also largely been observed in individuals at high risk for psychosis (i.e., familial risk or high clinical risk) (141, 142). More specifically, abnormal functional activation in risk groups has been reported in the prefrontal cortex, anterior cingulate cortex, amygdala, and temporal cortex (141, 142). Notably, also reduced amygdala volumes have been found in individuals at risk of psychosis (137, 142, 143), emphasizing the role of amygdala alteration as a potential liability to developing primary psychosis in those risk groups.

#### EEG Research

As the occipito-temporally recorded N170 ERP component is regarded as the primary component of face processing, the majority of ERP research on facial processing in primary psychosis has focused on this component (63, 144). A smaller N170 amplitude in individuals with primary psychosis compared with controls when processing expressive faces has been reported (58, 63, 145), and this effect seems to be independent of method of component extraction (mean vs. peak amplitude) or task requirements. Similarly, individuals with primary psychosis display smaller N250 and P300 amplitudes than healthy controls (63, 145).

A recent review by Earls et al. (144) suggested that N170 alterations in facial expression processing in primary psychosis may however stem from deficits in earlier visual processing, as for instance indexed by the P100. Indeed, also the P100 component is sensitive to facial stimuli and already shows emotional modulation. Individuals with primary psychosis display reduced P100 amplitudes when processing facial stimuli, indicating early sensory processing deficits. This effect, however, seems to depend on the emotional valence of the faces, as the difference between primary psychosis and controls was limited to neutral and happy faces, not fearful faces (144). Interestingly, a recent study revealed selectively decreased P100 amplitudes in individuals with negative schizotypy for all facial expressions, while there were no group differences in N170 amplitude. This finding underscores the early visual processing impairment in primary psychosis and thereby questions to what extent neural alterations are specific for expressive faces (146).

In line with the fMRI findings, to date, it is unclear whether EEG atypicalities in primary psychosis are specific to the processing of expressive faces or whether they arise when processing faces in general (63). In this regard, Murashko and Shmukler (64) and Shah et al. (145) reported reduced N170 amplitudes in individuals with primary psychosis when processing neutral faces, and this N170 amplitude to nonemotional faces also correlated with social functioning.

Altogether, EEG research shows robust alterations in face processing in primary psychosis. The high temporal resolution of EEG indicates that face processing impairments in primary psychosis are evident in the earliest ERP components and persist throughout processing (63). Yet, in line with the fMRI findings, it is questionable to what extent these alterations are specific to facial expression processing and do not merely result from altered face processing *per se*. To dissociate these alternatives, EEG paradigms directly contrasting neutral and emotional facial expressions are needed. A particularly promising approach for this would be the administration of a fast periodic visual stimulation oddball frequency-tagging paradigm, as has been applied in ASD (124, 125). Thus far, this method has not been applied in primary psychosis, but it was recently administered in patients with velocardiofacial (22q11.2 deletion) syndrome, which is a well-known high-risk group for psychosis (147). Interestingly, this study revealed significantly reduced emotion discrimination responses in the patient group (in particular for anger, disgust, and sadness) while showing slightly increased general visual face processing responses. Moreover, the neural response magnitude to expression changes was inversely associated with the severity of positive symptoms, pointing to a potential endophenotype and/or biomarker for psychosis risk (147). In this regard, this study strongly points toward emotion-specific facial processing impairments in psychosis-prone individuals while also echoing the findings of increased neural salience of faces *per se*.

A large number of studies have evaluated facial expression processing in primary psychosis using either fMRI or EEG, thereby contributing to our understanding of the neural nature of facial expression processing difficulties in this population. Nonetheless, multimodal imaging combining both methodologies is required to provide a deeper understanding of these mechanisms (148).

## The Impact of Adverse Childhood Events on Facial Expression Processing

### ACE Alter the Broader Face Processing Network

Face perception is in part an innate process but is also guided by visual learning and extensive social experiences (28, 149). As pointed out by the social motivation theory of ASD, early-onset impairments in social attention and social reward may initiate a developmental cascade that may ultimately deprive children of adequate social learning experiences, thereby also impacting on the neural sensitivity for facial expression processing (100). In a similar vein, extreme environmental factors, such as ACE, can disrupt these normative developmental experiences and can have detrimental effects on the neural basis of facial expression processing (29). Especially, due to the prolonged maturation of brain areas involved in the face processing network, these social brain regions are particularly vulnerable for the impact of stress during childhood (150–152).

These alterations in neurocognitive systems are hypothesized to be adaptive for survival in the face of adversity (151, 152). More specifically, according to the latent vulnerability theory, children exposed to adverse experiences show heightened neurocognitive vigilance to threat, including the processing of threatening social cues such as angry facial expressions (153). Whereas this hypervigilance may be adaptive in early at-risk environments, it may lead to psychiatric difficulties later in life (151–153).

In the past decades, research has focused on the impact of ACE on brain development (154, 155), and several structural and functional changes have been identified in individuals with a history of ACE. Structural changes partially depend on the age at ACE exposure (152) and include reduced brain volume in hippocampus, anterior cingulate, dorsolateral prefrontal cortex, and orbitofrontal cortex, amygdala alterations, and reduced white matter fiber tract integrity (151, 152). As can be expected based on the psychological impact of ACE and the involved structural alterations, ACE may alter behavioral face processing performance. More specifically, a majority of studies report deficits in facial expression recognition of both positive and negative emotions in children with a history of ACE. Furthermore, individuals with ACE exposure are more reactive toward negative expressions, especially anger, as they require lower levels of emotional intensity to recognize anger (156).

In terms of functional alterations, the most consistent observation is the increased responsivity of the amygdala in individuals with a history of ACE. Amygdala hyperactivity in response to expressive faces, especially threatening faces, has consistently been found in both children and adults with a history of ACE (157, 158). This finding is independent from psychiatric diagnosis, indicating that this hyperactivity may be inherent to the experience of adversity in itself (151, 152). Similar to behavioral studies, this hyperactivity suggests increased awareness for social threats and emotional sensitivity.

A recent meta-analysis by Hein and Monk (150) revealed significantly increased activation in the bilateral amygdala, right superior temporal gyrus, bilateral parahippocampal gyrus, and right insula in individuals with a history of ACE. Children and adolescents, but not adults, additionally show increased activity in the left lentiform nucleus and globus pallidus. In line with the behavioral studies indicating hypervigilance for negative or threatening emotions, this increased activation might facilitate rapid identification of threatening stimuli (150). Focusing specifically on individuals with a history of neglect, a recent review by Doretto and Scivoletto (29) revealed amygdala hyperactivity when processing fearful, angry, and sad faces, as well as greater hippocampal and ventromedial prefrontal cortex activation in response to fearful faces.

Pertaining to functional connectivity, increased functional communication in the fronto-limbic circuitry was reported in adults with a history of ACE performing an emotion-matching task. In particular, increased connectivity of the amygdala toward the orbitofrontal cortex, ventrolateral prefrontal cortex, dorsomedial, and dorsolateral prefrontal cortex, and hippocampus has been shown (159).

EEG research also shows substantial differences in individuals exposed to ACE compared with unaffected control populations. On the one hand, reduced face-specific ERPs (i.e., P100, N170, and P400) are observed in children exposed to extreme psychosocial deprivation in institutional rearing when processing faces, regardless of the displayed expression (157). One longitudinal study assessed facial expression processing in institutionalized vs. family-reared children at baseline (i.e., between the age of 5 and 31 months) and 30 and 42 months old (160). Institutionalized children displayed reduced ERPs at baseline compared with family-reared children. Furthermore, children that are placed into foster care following institutionalization had intermediate ERP amplitudes and latencies compared with institutionalized and family-reared children at 30 and 42 months. On the other hand, increased ERP amplitudes, indicative of social hypervigilance, are observed in individuals with an effective history of ACE. A review by da Silva Ferreira et al. (156) revealed that a majority of studies found higher ERP amplitudes in response to angry faces in maltreated children. Some studies in individuals with a history of ACE also revealed larger N170 amplitudes when processing any type of expressive faces. Remarkably, this increased amplitude was not limited to negative emotions, indicating increased vigilance during facial expression processing irrespective of the valence of the facial expression (161). Similarly, adults with a history of interpersonal childhood adversity fail to show a reduced N170 amplitude toward subconscious happy relative to angry faces, again suggesting hypervigilance toward expressive faces and a failure to differentiate between threatening and nonthreatening stimuli (162).

Altogether, the findings reported above indicate robust altered facial expression processing in children, adolescents, and adults with a history of ACE. Considering the characteristic general facial expression processing difficulties in individuals with ASD and primary psychosis, and given the substantially increased prevalence of ACE in these particular populations, we may contend that a history of ACE in these individuals might even further hamper and complicate face processing skills in these populations and add to the reported inconsistencies in the literature. In the following, we will briefly explore the sparse literature discussing the impact of ACE on social processing in individuals with ASD and/or primary psychosis.

### Increased Prevalence of ACE in Individuals With ASD

Youth with developmental disabilities, such as ASD, have 1.5 to 3 times more chance of encountering ACE than their peers (17) and are twice as likely to experience four or more different types of ACE (14). Especially their sociocommunicative characteristics (e.g., difficulties with emotional insight and information processing, mental rigidity, etc.), as well as their predisposition for experiencing large levels of familial stress, make them more susceptible to ACE (18). Despite the increased vulnerability of individuals with ASD for trauma, literature on the association between ASD and ACE is still in its infancy (18, 163). One factor that may contribute to the lack of studies investigating this association is the overlap in diagnostic criteria for ASD and posttraumatic stress disorder (1), hampering the identification of trauma in individuals with ASD (17, 163). For example, individuals who experienced ACE may struggle with social interactions, may display hyperarousal to sensory stimuli, and may show circumscribed interests and reduced affect, which are all core symptoms of ASD. In light of these similarities, it is not surprising that a history of ACE has been found to be related to a delay in ASD diagnosis (14).

Probably because this research field is still emerging, we could not find any study investigating behavioral or neural facial expression processing in individuals with ASD and a history of ACE. Nevertheless, as pointed out in the discussion, integration of both research fields might be highly elucidating, in particular because investigation of the counteracting effects on facial expression processing (e.g., amygdala hypoactivation in ASD and hyperactivation in ACE) could possibly clarify some of the existing inconsistencies in the field.

### The Impact of Comorbid ACE and Primary Psychosis on Facial Expression Processing

Primary psychosis develops as a result of a complex interplay between genetic and environmental factors, including childhood adversity (164). ACE are associated with a two- to fourfold increased risk of primary psychosis, and the prevalence of ACE in individuals with primary psychosis is consistently higher than in the general population (15, 165, 166). Furthermore, exposure to ACE is higher and more severe in individuals at ultra-high risk of psychosis, with prevalence rates ranging from 54 to over 90% (167). Several studies have shown a dose–response relationship between the severity of ACE and the severity of (positive) psychotic symptoms such as hallucinations and delusions, suggesting a causal association between ACE and primary psychosis (15, 16, 166, 168).

Despite consistent evidence of the impact of ACE on facial expression processing, despite the large comorbidity between ACE and primary psychosis, and despite the potential confounder of this comorbidity for findings on facial expression processing in the psychosis literature, only a limited number of studies have assessed facial expression processing in individuals with psychosis and a history of ACE. At the behavioral level, Mrizak et al. (169) showed that adults with primary psychosis and a high exposure to ACE perform significantly worse on facial emotion recognition tasks compared with adults with primary psychosis without a history of ACE. Furthermore, the authors suggested that the type of abuse could be associated with specific emotion recognition deficits. In particular, in this study, sexual abuse was associated with poor recognition of anger and disgust, and emotional abuse and physical neglect were associated with poor recognition of happy and sad faces (169).

Aas et al. (170) applied fMRI to assess neural activation during a facial expression processing task in individuals with primary psychosis with vs. without ACE exposure. Patients with higher ACE exposure displayed a relatively stronger neural activation in response to negative expressions (i.e., angry and fearful faces) in the right lateral occipital cortex (i.e., fusiform gyrus), middle temporal gyrus, angular gyrus, and supramarginal gyrus. As such, individuals with primary psychosis and a high ACE exposure might show an activation pattern closer to that reported in healthy individuals, yet no comparison with healthy controls could be made in this study. Nonetheless, this difference in neural activation between individuals with high and low ACE was associated with poorer daily life functioning. Behaviorally, individuals with primary psychosis and high ACE exposure evaluated negative faces as more negative, and positive faces as less positive than those with low ACE exposure (170).

Pertaining to EEG findings, Gong et al. (146) presented expressive (i.e., happy, angry, fearful, and disgusted) and neutral faces to individuals with high and low levels of negative schizotypy with and without a history of ACE using a dot-probe task. Individuals with negative schizotypy and a history of ACE displayed a longer P100 latency independent of emotion, indicating a general dysfunction of the visual pathway. This study found no differences in P100 amplitude nor in N170 amplitude and latency.

In sum, ACE seem to alter facial expression processing impairment in individuals with primary psychosis. On the one hand, Aas et al. (170) reported increased neural activation in response to negative faces in individuals with primary psychosis and a history of ACE. On the other hand, individuals with negative schizotypy and ACE exposure have a longer P100 latency to faces, revealing a general dysfunction in visual processing (146). More research is needed to elucidate the relation between ACE, primary psychosis, and facial expression processing.

## Discussion

The aim of this narrative review was twofold: we aimed to (i) provide a concise overview of the currently reported neural commonalities and differences in individuals with ASD and primary psychosis when processing expressive faces and (ii) explore and provide insight into how ACE might influence facial expression processing in ASD and primary psychosis.

### Altered Neural Facial Expression Processing in ASD and Primary Psychosis

Although the neuroimaging literature on facial expression processing in ASD appeared to entail many more inconsistencies in comparison with the literature on primary psychosis, both disorders show altered neural facial expression processing as compared with healthy controls. Indeed, individuals with ASD, as well as individuals with primary psychosis, activate the same brain regions as healthy controls during facial expression processing, albeit to a significantly lesser and/or different extent (133–135, 171). Overall, when compared with healthy controls, individuals with ASD and individuals with primary psychosis mainly display altered activation in the fusiform gyrus and amygdala as well as altered connectivity among the broader face processing network, probably indicating reduced facial expression processing abilities.

Reduced activation in the fusiform gyrus—and indirectly also atypical amygdala functioning—might be associated with delayed or reduced N170 responses, often reported in individuals with ASD (61) or primary psychosis (58, 63, 145), respectively, as this ERP component is mostly recorded over occipito-temporal sites where the fusiform gyrus is located. Studies assessing facial expression processing using multimodal imaging (i.e., integrating imaging methodologies) are invaluable to enhance our understanding of the neural underpinnings of facial expression processing in ASD and primary psychosis.

In addition to reduced activity in the fusiform gyrus, also altered activity of the amygdala is generally found in ASD and primary psychosis. According to the amygdala theory of autism (95), atypicalities in the amygdala (such as the observed hypoactivation) are at the root of the social deficits characteristic for ASD. Similarly, the severity of blunted affect (i.e., one of the negative symptoms) has been found to be associated with amygdala activation during facial expression processing in individuals with primary psychosis (172). However, the reduced amygdala activation in primary psychosis vs. healthy controls is mostly found when expressive faces are contrasted to neutral faces (136, 137, 139) and potentially results rather from an enhanced brain response to neutral faces, than a decreased response to expressive faces (140). This aligns with the aberrant salience hypothesis, stating that salience is attributed to irrelevant stimuli (here, neutral faces), which then attract more attention (138). Whereas individuals with primary psychosis might thus attribute too much salience to socially irrelevant stimuli, thereby masking the salience of truly relevant social stimuli, individuals with ASD show an overall reduced tendency to orient to social stimuli [social motivation theory; (100)].

### Experiences Tuning the Brain Toward Social Signals: the Case of ASD and Primary Psychosis

As the progressive tuning of the neural system involved in facial expression processing (30, 42) is enhanced by social experiences (173), deprivation of social interaction might hamper further specialization of this system. Hence, in ASD, the reduced tendency to orient to social stimuli and/or to participate in social interactions (174) might hamper acquirement of the facial expression processing experiences necessary for typical maturity of these abilities and of this neural system. Likewise, in primary psychosis, the progressive decline in face processing abilities with increasing age and increasing illness duration [(64, 132); but see (27)] may partially result from the progressive social withdrawal typical for this population.

Additionally, both in ASD and primary psychosis, the maturation of facial expression processing abilities—and, thus, the activation of the corresponding brain regions—might be hampered by deficits in general visual perception (175), as difficulties in emotion processing may occur when one fails to inspect the most relevant facial cues (176). Indeed, similar to ASD [e.g., (177, 178)], neuroimaging and eye-tracking studies in individuals at risk of psychosis and those with primary psychosis have revealed more local and fragmented processing of faces as well as avoidance of crucial face areas such as the eyes, nose, and mouth [e.g., (179)].

### Experiences Tuning the Brain Toward Social Signals: the Case of ACE

With the exception of studies assessing the impact of neglect and extreme social deprivation, which results in a uniformly blunted response for all socioemotional cues (157, 160), the majority of studies investigating the impact of ACE clearly show an increased sensitivity for facial expressions, in particular negative emotions such as anger and fear (156, 161). At the neural level, this hypervigilance for social cues signaling a potential threat seems to be driven by a hyperactive amygdala, which may continuously alert and arouse the broader social brain through a hyperexpressed functional connectivity (150, 151). As a result, and somewhat in line with the aberrant salience hypothesis in primary psychosis (138), too much undifferentiated salience is attributed to any social signal, thereby impeding a more thorough and fine-grained discrimination between social cues. However, to our knowledge, only one study assessed facial expression processing in individuals exposed to adversity and followed them up longitudinally (160). Longitudinal studies are crucial to draw strong conclusions regarding the causal effects of ACE on facial expression processing.

The robust effects of ACE on facial expression processing may be reversible to some extent if early intervention, such as good rearing circumstances, is applied. For instance, children that were reared in institutions but eventually placed in good foster care had intermediate P100, N170, and P400 amplitudes as compared with institutionalized and family-reared children (157, 160). Likewise, there is emerging but still highly conflicting evidence for the neuroplasticity of facial expression processing abilities in ASD and primary psychosis via social cognition training or via targeted expression processing [(180–184); but see (185)]. Yet, it remains questionable to what extent this training may generalize to daily life face processing and may effectively improve social functioning outcomes.

### Need for Integrative Studies Across Multiple Clinical Conditions

As stated previously, ASD and psychosis represent spectrum disorders with a considerable epidemiological, phenotypical, and neural overlap. Likewise, individuals with ASD, as well as individuals with primary psychosis, have a much higher chance to encounter ACE as compared with their peers. Against this background, it seems imperative to design studies that explicitly contrast the three clinical conditions or that at least control for comorbidity and/or presence of dimensional (sub)clinical characteristics. This is *a fortiori* the case for facial expression processing research, as it yields atypical findings among the three populations, but with distinctive flavors in each of the particular populations. Such an approach might eventually also account for some of the inconsistencies and confound reported in the field.

Thus far, only very few studies directly contrasted facial expression processing in individuals with ASD vs. individuals with primary psychosis, or in individuals with primary psychosis with vs. without a history of ACE. To our knowledge, no face processing studies accounted for the presence of ACE history in individuals with ASD.

Direct comparisons of facial expression processing in samples with ASD vs. samples with primary psychosis are scarce. In general, individuals with primary psychosis tend to perform slightly better than individuals with ASD on behavioral face processing tasks, yet this group difference seems to decline with increasing age [(22, 175); but see a recent study by Pinkham et al. (186)]. Probably, this decreasing group difference with age can be understood as follows: on the one hand, because increasing age and thus illness duration may be associated with more severe impairment in primary psychosis; on the other hand, because more learning experiences due to increasing age may ultimately improve facial expression processing in adults with ASD.

The few studies investigating facial expression processing in individuals with primary psychosis with vs. without a history of ACE show mixed results. On the one hand, an enhanced neural processing of negative stimuli [i.e., increased brain activity; (170)] has been reported, but this does not seem to translate in improved behavioral processing. Indeed, behaviorally, individuals with primary psychosis, and a history of ACE perform significantly worse on emotion recognition tasks than adults with primary psychosis without ACE exposure (169). This pattern may suggest that ACE may further boost the general aberrant salience in primary psychosis, thereby causing overarousal and impeding a differentiated behavioral facial expression processing performance. On the other hand, the single study assessing ERPs in individuals with primary psychosis and a history of ACE revealed a longer P100 latency when viewing expressive and neutral faces, suggesting a larger deficit in visual processing (146).

Altogether, these few studies may suggest an additional aggravating effect of ACE on facial expression processing impairments in primary psychosis. Thus far, no studies investigated facial expression processing in ASD while accounting for individual differences in ACE experiences. However, given the opposite and possibly counteracting pattern of neural atypicalities, with reduced neural responses toward especially negative emotional expressions in ASD and enhanced neural responses toward especially negative and threatening facial expressions in individuals with a history of ACE, this integrative approach would be particularly informative. Eventually, it may possibly account for the large interindividual heterogeneity in the autism population and may explain (part of) the observed inconsistencies across studies.

Preferentially, these future studies should not only incorporate interindividual variability and comorbidity of the target populations but should also account for variability in research methods and designs, as these may also partially account for the differences and inconsistencies encountered in the neuroimaging literature. Task demands, for example, have been found to influence neural responses. Indeed, differences in facial expression processing in healthy controls vs. individuals with ASD (75) or primary psychosis (179) have most frequently been reported when tasks are more demanding, or when tasks involve implicit automatic processing instead of explicit facial expression processing (102, 108, 136). Furthermore, stimulus characteristics, such as the intensity of the expressions (83, 96), familiarity of the faces (109), or the use of static vs. dynamic expressions (54), have also been found to modulate the neural responses and may therefore differentially impact on individuals with different clinical status.

### The Unfulfilled Search for a Selective Biomarker of Facial Expression Processing Impairments and/or Socioemotional Dysfunctioning

Overall, individuals with ASD or primary psychosis display impairments in facial expression processing, both at a behavioral and at a neural level. This has led researchers to suggest that impaired facial expression processing may be a vulnerability marker for primary psychosis (27) or ASD (61). In the past decades, greater affordability and accessibility of noninvasive brain imaging techniques have led to an intense quest for an objective brain-based biomarker that could predict risk, support clinical diagnosis, or monitor treatment effects (138, 185, 187). This urge is particularly strong in the field of neurodevelopmental disorders, such as ASD, in which standardized behavioral assessment options are often limited and access to clinical expertise is not always readily available.

Against this background, the N170 ERP component has often been put forward as a promising biomarker, both within the psychosis literature (27, 58) and within the ASD research field (61), and often largely independent from each other. Multiple reports have indeed suggested that N170 amplitudes are generally smaller and latencies slower regardless of psychiatric disorder. Accordingly, the N170 component has been proposed to index impairments in the extraction of facial expression information, an impairment that is common across disorders and could be related to social functioning (58).

However, it should be noted that the N170 is a very rough index that is not specific for facial expression processing and that lacks specificity, objectivity, sensitivity, and reliability, as pointed out in a number of recent reports (121, 147, 185, 188). Most importantly, the N170 does not consistently categorize individuals and does not measure a specific impairment (facial expression processing) related to a specific clinical profile (e.g., ASD). Moreover, disentangling the specific neural response to the facial expression from the general neural face processing response is challenging, especially since deficits in general (neutral) face processing have also been reported in both ASD (177, 189) and primary psychosis (64, 145, 179). Accordingly, and in spite of many studies claiming otherwise [e.g., (58, 61)], it is highly questionable whether the N170 may ever fulfill its promise of being a sensitive biomarker for aberrant socioemotional sensitivity and definitely not for disorder-specific dysfunction [e.g., (121, 185, 190)].

In the past years, an alternative EEG approach, called fast periodic visual stimulation frequency-tagging EEG, has been put forward, yielding promising findings pinpointing selective facial expression processing impairments in individuals with ASD (124, 125) and primary psychosis (147). This novel approach reveals an objective, selective, reliable, and behavior-free signature of impaired visual coding of facial expression, implicitly quantified from brain activity with high signal-to-noise ratio at the individual subject level. Given the strength of the obtained effects, the implicit nature, the rapid application, and straightforward analysis, this novel tool may open avenues for clinical practice, potentially providing a biomarker for individual assessment of aberrant socioemotional sensitivity across syndrome boundaries.

Future integrative multipopulation studies looking into the associations of aberrant facial expression processing among ASD and primary psychosis, and the modulating impact of adverse childhood events, might benefit of incorporating this pioneering frequency-tagging EEG approach.

## Limitations

With this narrative review, we hope to have identified the gaps and inconsistencies in the existing literature on facial expression processing in ASD and primary psychosis, and the possible impact of ACE upon this, which could encourage and inspire future researchers to further investigate this topic. While we aimed to cover this field in a comprehensive manner, the possibility of a subjective selection bias cannot be fully excluded. In addition, given the extensiveness of the literature on facial expression processing in ASD and primary psychosis, we specifically focused on behavioral emotion processing and on neural activity and connectivity patterns as investigated via EEG and fMRI.

## Author Contributions

CS and SV wrote the first draft of the manuscript. RW and BB provided thorough feedback to the first draft. All authors contributed to manuscript revision and approved the submitted version.

## Conflict of Interest

The authors declare that the research was conducted in the absence of any commercial or financial relationships that could be construed as a potential conflict of interest.
